# Population genomics in neglected malaria parasites

**DOI:** 10.3389/fmicb.2022.984394

**Published:** 2022-09-08

**Authors:** Awtum M. Brashear, Liwang Cui

**Affiliations:** Department of Internal Medicine, Morsani College of Medicine, University of South Florida, Tampa, FL, United States

**Keywords:** malaria, neglected, genomics, population genomics, vivax malaria

## Abstract

Malaria elimination includes neglected human malaria parasites *Plasmodium vivax, Plasmodium ovale* spp., and *Plasmodium malariae*. Biological features such as association with low-density infection and the formation of hypnozoites responsible for relapse make their elimination challenging. Studies on these parasites rely primarily on clinical samples due to the lack of long-term culture techniques. With improved methods to enrich parasite DNA from clinical samples, whole-genome sequencing of the neglected malaria parasites has gained increasing popularity. Population genomics of more than 2200 *P. vivax* global isolates has improved our knowledge of parasite biology and host-parasite interactions, identified vaccine targets and potential drug resistance markers, and provided a new way to track parasite migration and introduction and monitor the evolutionary response of local populations to elimination efforts. Here, we review advances in population genomics for neglected malaria parasites, discuss how the rich genomic information is being used to understand parasite biology and epidemiology, and explore opportunities for the applications of malaria genomic data in malaria elimination practice.

## Introduction

**FIGURE 1:**
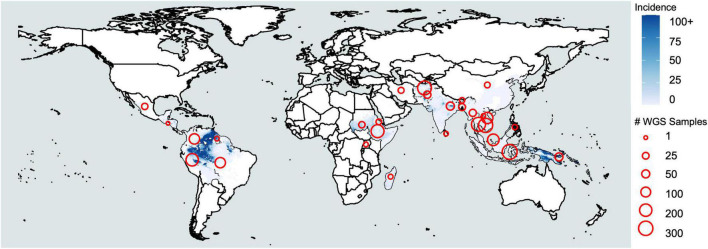
*Plasmodium vivax* incidence and whole-genome sequencing. Coloration represents incidence per 1000 based on raster data from the Malaria Atlas Project (https://malariaatlas.org/malaria-burden/) in 2017, the most recent year for which data is available. Red circles represent whole-genome sequencing samples collected by country based on Supplementary Table 1.

Malaria is caused by infection with protozoan parasites from the genus *Plasmodium*. Five species are commonly accepted as human malaria parasites: *Plasmodium falciparum*, *Plasmodium vivax*, *Plasmodium malariae*, *Plasmodium ovale curtisi*, and *Plasmodium ovale wallikeri*. The latter two are recognized as morphologically indistinguishable, sympatric, but non-recombining species only in the last decade (Sutherland et al., 2010) and are frequently combined as *P. ovale* spp. Additionally, several species infecting non-human primates occasionally cause zoonotic malaria in humans—including at least *P. knowlesi* and *P. cynomolgi* in Asia (Antinori et al., 2013; Raja et al., 2020) and *P. simium* in South America (Brasil et al., 2017). The occurrence of the zoonotic malaria reflects the distribution of their primary hosts. Among the human malaria parasites, *P. falciparum* is associated with most malaria-related deaths (Gething et al., 2016), whereas *P. vivax* is the most widespread and dominant species outside of Africa (Howes et al., 2016; Figure 1). It is estimated there were 7.5 million vivax malaria cases globally in 2018, more than half of which occurred in Southeast Asia (World Health Organization [WHO], 2019). In comparison, *P. malariae* and *P. ovale* spp. are distributed globally, but the incidence is low. Though generally less virulent than *P. falciparum*, *P. vivax* can also cause severe pathology, and recurrent episodes greatly increase morbidity (Anstey et al., 2009; Baird, 2013). *P. malariae* and *P. ovale* spp. are sporadically associated with severe clinical manifestations such as anemia, acute respiratory distress, and renal failure (Lau et al., 2013; Langford et al., 2015; Kotepui et al., 2020). Resources for malaria control and research are largely committed to *P. falciparum*, while the neglected human malaria parasites are understudied, resulting in significant gaps in our understanding of their fundamental biology (Mueller et al., 2009). We refer to these non-falciparum human malaria parasites as “neglected malaria parasites.”

**FIGURE 2:**
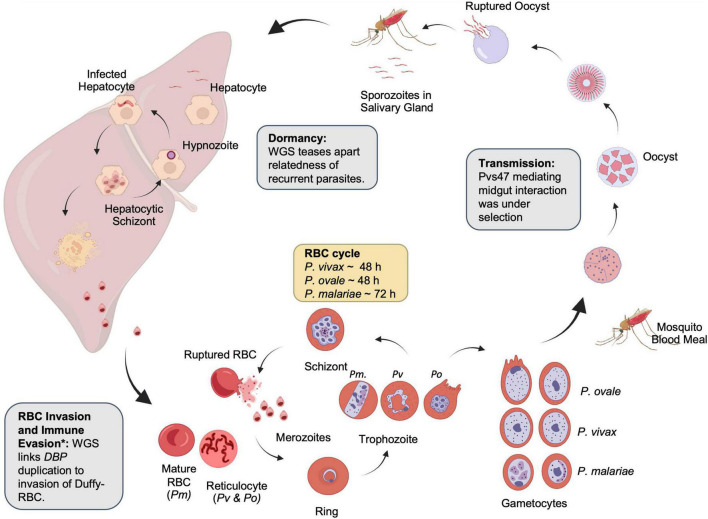
Lifecycle of non-falciparum malaria parasites. Examples of the contribution of whole-genome sequencing to parasite biology are shown in boxes. Created with BioRender.com. *The identification of the relationship between duffy-binding protein duplication and immune evasion was identified with qPCR.

All malaria parasites share a similar life cycle involving a vertebrate intermediate host such as a human and a definitive host such as an *Anopheles* mosquito (Figure 2). The neglected malaria parasites have several distinctive features favoring and sustaining transmission. *P. vivax* and *P. ovale* spp. invade young reticulocytes (Simpson et al., 1999; Lim et al., 2016), leading to low-density infections. *P. malariae* is also associated with low-density infections in part due to a preference for older red blood cells (RBCs), production of fewer merozoites per schizont (average of 8), and an extended erythrocytic cycle (72 h) (Collins and Jeffery, 2007). Low-density infections are often missed by routine diagnostic methods such as microscopy and rapid diagnostic tests and may constitute an adaptation of the parasites for sustained transmission (Sutherland, 2016). Gametocytes emerge sooner in *P. vivax* than in *P. falciparum*, resulting in more efficient transmission, even among mixed infections (Balasubramanian et al., 2020). Finally, *P. vivax* and *P. ovale* spp. form dormant hypnozoites in the liver, which can relapse months or years later (White and Imwong, 2012). These characteristics bestow the neglected malaria parasites the ability to withstand traditional control measures designed for *P. falciparum*, with their prevalence or proportions often increased in areas of co-endemicity with *P. falciparum* (Cotter et al., 2013; Geng et al., 2019; Yman et al., 2019). Since neglected malaria parasites must also be eliminated (Baird, 2010; Lover et al., 2018), a better understanding of their biology, epidemiology, and evolution is needed.

Advances in next-generation sequencing (NGS) technologies have enabled whole-genome sequencing (WGS) of a large number of clinical isolates of malaria parasites, allowing the study of parasite populations at the genomic level – population genomics. However, there have been significant barriers to using the WGS technology within the neglected malaria species. The most notable is the reliance on clinical samples, which often have low parasitemia and contamination with human genomic DNA. Since the human genome (∼3000 Mb) is about 100 times larger than the malaria parasite genome (25–35 Mb), the problem associated with the human genome is obvious. Therefore, leukocyte depletion using filtration methods (e.g., CF-11 columns or commercial filters) is instrumental for obtaining high-quality parasite genomes (Auburn et al., 2011, 2013; Venkatesan et al., 2012; Rutledge et al., 2017b; Brashear et al., 2020b). Additional whole-genome amplification (WGA) may be necessary to further increase the total parasite DNA (Dharia et al., 2010; Brashear et al., 2019). For archived samples (often preserved as dried blood spots on filter paper) or samples collected in endemic areas where on-site leukocyte depletion cannot be performed, parasite DNA needs to be enriched. An in-solution hybridization method called hybrid selection or genome capture (Melnikov et al., 2011) has been used with genome-wide synthetic biotinylated RNA baits (Bright et al., 2012, 2014) or synthetic oligos (Hupalo et al., 2016), but the synthesis of baits can be costly, and this procedure is prone to bias as regions with higher GC content will hybridize more efficiently. Recently, selective WGA (SWGA), which uses specifically-designed small sets of short primers specific to the genome of interest to amplify the target genome preferentially, has become a cost-effective and robust procedure suited to enrich *Plasmodium* DNA (Leichty and Brisson, 2014). SWGA protocols have been designed for *P. falciparum*, *P. vivax*, and *P. malariae* (Oyola et al., 2016; Cowell et al., 2017; Ibrahim et al., 2020). Another innovative method, combining cell sorting with WGA to obtain highly accurate parasite genome information from single parasite-infected erythrocytes (Nair et al., 2014), is particularly suited for studying within-host variations of malaria infections from hyperendemic regions and teasing out the genetic complexities and relatedness of parasites within single hosts (Nkhoma et al., 2020). Here we review the recent progress in population genomics of neglected parasites focusing on *P. vivax* due to the breadth of literature available and discuss how the genomic data can be exploited to understand parasite biology and guide malaria elimination efforts.

## Establishing reference genomes

In terms of accuracy and completeness, the *P. falciparum* 3D7 genome is the most complete reference *Plasmodium* genome (Gardner et al., 2002), and it has been further validated using the PacBio long-read sequencing technology (Otto et al., 2018). However, *P. falciparum* genomes are not an ideal reference for other medically important malaria parasites. The first draft of the *P. vivax* Sal-I nuclear genome consisted of over 2500 scaffolds totaling ∼26.8 Mb, of which ∼22.6 Mb were assigned to the 14 chromosomes (Carlton et al., 2008). Shortly after, the NGS technology enabled a multitude of whole-genome assemblies, including four geographically divergent *P. vivax* strains from North Korea, India, Brazil, and Mauritania (Neafsey et al., 2012). *De novo* assembly of a Cambodian *P. vivax* isolate highlighted shortcomings of the Sal-I assembly by identifying nearly 800 unaccounted-for genes (Hester et al., 2013). The 2016 *P. vivax* PvP01 genome from Papua New Guinea (PNG) dramatically improved assembly quality, reflected in the substantially reduced number of unassigned contigs and better resolved subtelomeric regions where there were nearly four times as many hypervariable *pir* genes as identified from the Sal-I assembly (Auburn et al., 2016a). The recently assembled new reference genomes of *P. vivax* strains from the China-Myanmar border region were of comparable quality to the PvP01 genome (Brashear et al., 2020b). With this new information, the *P. vivax* genome is estimated at ∼30 Mb. The expansion of subtelomeric gene families likely explains the increased genome sizes of the neglected malaria parasites compared to ∼23 Mb of the *P. falciparum* genome. Genomes for two *P. ovale curtisi* strains, two *P. ovale wallikeri* strains, and one *P. malariae* strain were first reported in 2016 (Ansari et al., 2016). The two *P. ovale wallikeri* and one *P. ovale curtisi* isolates were recent clinical samples from travelers who were infected in Gabon and Nigeria, respectively, while the *P. malariae* (Uganda) and one *P. ovale curtisi* (Nigeria) were historic samples isolated in 1974 and 1977, respectively, and passaged through chimpanzees. Subsequently, a high-quality genome assembly of a *P. malariae* strain from Uganda using long-read sequencing technology and draft genomes of *P. ovale curtisi* (from Ghana) and *P. ovale wallikeri* (from Cameroon) assembled from *P. falciparum* co-infections were reported (Rutledge et al., 2017a). Compared with *P. vivax* and *P. falciparum*, the genome sizes were much larger for *P. ovale* spp. (33–38 Mb) and *P. malariae* (∼34 Mb).

Fragmented reference genome assemblies can pose problems for analyses that rely on highly accurate alignment to reference genomes. Programs that detect insertions or deletions (indels) are especially affected as they depend on discordant read alignment and read depth (Abyzov et al., 2011; Layer et al., 2014; Beghain et al., 2016), both confounded by mis-mapping. Since the parasite genomic DNA of non-falciparum species has to be obtained almost exclusively from clinical samples, genome assemblies for these species have mostly relied on short-read sequencing, which is ineffective at resolving the abundant repetitive regions. The limitations of short-read sequencing may soon be overcome with long-read technologies such as the PacBio and Oxford Nanopore sequencing technologies, as demonstrated with recent successful applications in improving *Plasmodium* genome assemblies (Vembar et al., 2016; Rutledge et al., 2017a; Otto et al., 2018). Fortunately, incomplete reference genomes should not substantially hinder traditional population genomic studies of the neglected malaria parasites—wherein sequence reads of field isolates are typically aligned to reference genomes for calling single nucleotide polymorphisms (SNPs)—given the high-level synteny and conservation of the core genomes.

Notably, the gene content and quality of annotation are continually improving. Despite *P. falciparum* being the most well-annotated species, the most recent version of PlasmoDB (Update 57) included updates of 223 *P. falciparum* genes and less than 60 updated genes for any other human or primate *Plasmodium* species (PlasmoDB, 2022). Meanwhile, certain annotation resources, such as the Malaria Parasite Metabolic Pathways (MPMP), are exclusively available for *P. falciparum* (Ginsburg and Abdel-Haleem, 2016). Researchers often use studies performed in orthologs from *P. falciparum* or a rodent model to annotate the genomes of neglected malaria parasites, although they differ substantially. It was found that *P. vivax, P. malariae, P. knowlesi, P. ovale curtisi*, and *P. ovale wallikeri* shared just 3429 ortholog groups, with *P. ovale wallikeri* having 1036 unique ortholog groups (Ansari et al., 2016). The expansion of subtelomeric genes in non-falciparum malaria parasites is something noted in many genomic assemblies, but very little is known about the purpose of the additional gene family members. Surfin or *STP1* genes, for example, have few members in *P. vivax* and *P. falciparum* and are lacking in *P. knowlesi*, but have dozens to hundreds of members in *P. malariae* and *P. ovale* spp. (Ansari et al., 2016). Likewise, PfEMP1 and SICAvar proteins are present in *P. falciparum* and *P. knowlesi*, respectively (Frech and Chen, 2013). Thus, the neglected parasites deserve special attention in genomics and gene annotations.

## Understanding parasite biology using genomics

Population genomics of *P. vivax* is instrumental for improving our knowledge about its distinct parasite biology, including reticulocyte preference, invasion of Duffy-positive RBCs, and relapse (Figure 2). The rarity of vivax malaria transmission in African populations has been postulated to be due to the absence of Duffy antigen/receptor for chemokines (DARC) gene expression, once considered a requirement for erythrocyte invasion. Confirmed cases of *P. vivax* parasites in Duffy-negative Africans and the potential spread of such parasites suggest that *P. vivax* may have evolved to invade Duffy-negative individuals (Menard et al., 2010; Gunalan et al., 2016; Kepple et al., 2021). Duplication of the *Duffy binding protein 1* (*pvdbp1*) gene was common in Malagasy *P. vivax* strains infecting Duffy-negative individuals (Menard et al., 2013). A very high proportion (>50%) of parasites with amplified *pvdbp1* gene was detected in Ethiopia, where ∼35% human population is Duffy negative (Hostetler et al., 2016; Pearson et al., 2016; Auburn et al., 2019; Lo et al., 2019; Ford et al., 2020). However, it is intriguing that parasites with amplified *pvdbp1* also reached modest levels (20–38%) in the Greater Mekong subregion (GMS), where nearly all local populations are Duffy positive (Hostetler et al., 2016). WGS analysis provided details of the Southeast Asian *pvdbp1* amplification events, revealing two types of duplication, the Cambodian-type and the Malagasy-type, and indicated independent evolution of *pvdbp1* duplication on the different genetic backgrounds (Hostetler et al., 2016; Auburn et al., 2019; Lo et al., 2019; Ford et al., 2020). Though the significance of *pvdbp1* expansion in the invasion of Duffy-negatives in Africa remains to be tested, *pvdbp1* amplification in Cambodian parasites was shown to result in increased *pvdbp1* mRNA levels and associated with the ability of the parasites to counteract host anti-PvDBP antibodies (Popovici et al., 2020).

Relapses account for a significant source of blood-stage infections for *P. vivax* and *P. ovale* spp. (Robinson et al., 2015). The phenotypes of relapse in *P. vivax* malaria are well characterized, but the mechanisms of hypnozoite formation and reactivation remain unknown (White and Imwong, 2012). Relapsing parasites often differ from the parasites in the initial acute infection (Chen et al., 2007; Imwong et al., 2007), but it is difficult and sometimes impossible to determine whether a recurrent blood-stage infection arises from recrudescence, relapse, or new infection. Amplicon deep sequencing of polymorphic antigens demonstrated the complexity of the initial blood-stage infections and provided a scheme to assign the probability of a recurrent infection as a relapse (Lin et al., 2015). WGS data offers additional power to dissect the relatedness of individual parasite clones within a blood-stage infection. In a confirmed case of multiple relapses in a patient who acquired the initial infection in Eritrea, WGS revealed that parasites from three relapse episodes were genetically related meiotic siblings (Bright et al., 2014). In one study of Peruvian *P. vivax*, WGS was used to examine 23 paired clinical samples from the same patients at the times of initial infection and recurrent infection after three primaquine regimens (Cowell et al., 2018). By comparing the degrees of Identity-by-Descent (IBD, Box 1) between the paired samples, these researchers identified 12 cases of homologous relapses (IBD > 98%) and three heterologous relapses with highly related parasites which could be meiotic siblings. In the same study, WGS allowed more accurate assignment of recurrent cases compared to that based on microsatellite genotyping data (Durand et al., 2014). Similarly, a study examining relapse dynamics was conducted in Cambodia in 20 *P. vivax* patients within 2 months after CQ treatment (Popovici et al., 2018). WGS of five paired samples and SNP typing of others confirmed the multiclonality nature of relapses, and the relapsing parasites showed various degrees of relatedness in IBD to the parasites present in the original infection, consistent with the higher endemicity level of the study area (Popovici et al., 2018, 2019). Environmental factors such as epidemiology impact the nature of recurrence, with more intensive transmission increasing the chance of heterologous relapses (Imwong et al., 2012). Recent models have incorporated microsatellite-based IBD data and time between initial and recurrent infections for estimating recurrent infections as either recrudescence, relapse, or reinfection (Taylor et al., 2019b). Applying this multi-factor principle to genomic data may be incredibly fruitful for classifying infections in the future.

**BOX 1. Identity-by-descent methodologies.**

One important technique empowered by WGS is Identity-by-Descent estimation. IBD segments are defined as being inherited from the same ancestor, so longer lengths of IBD segments can suggest a more recent divergence between two individuals (Shetty et al., 2019; Taylor et al., 2019a). Various tools allow researchers to infer relatedness *via* IBD inference using SNP data. HmmIBD benefits non-falciparum malaria species due to its built-in consideration of SNPs for distance rather than genetic time (Schaffner et al., 2018). This feature removes the necessity of a genetic map, which has not been standardized *via* hybrid crossing for non-falciparum species. IsoRelate (Henden et al., 2018) does require a genetic map which users may improvise from linkage disequilibrium data, but it can consider infections harboring multiple parasite strains. DEploid-IBD can infer the relatedness of multiple infecting strains (Zhu et al., 2018); however, reliance on a high-confidence reference haplotype panel is problematic for neglected species. It is important to note that while the proportion of two genomes that are in IBD is correlative with estimated relatedness, it is not a perfect indicator, and it can be biased by the chosen SNP sets. Therefore, comparing IBD across whole genome studies is not recommended (Taylor et al., 2019a).

## Understanding epidemiology from estimates of the complexity of infections

**FIGURE 3:**
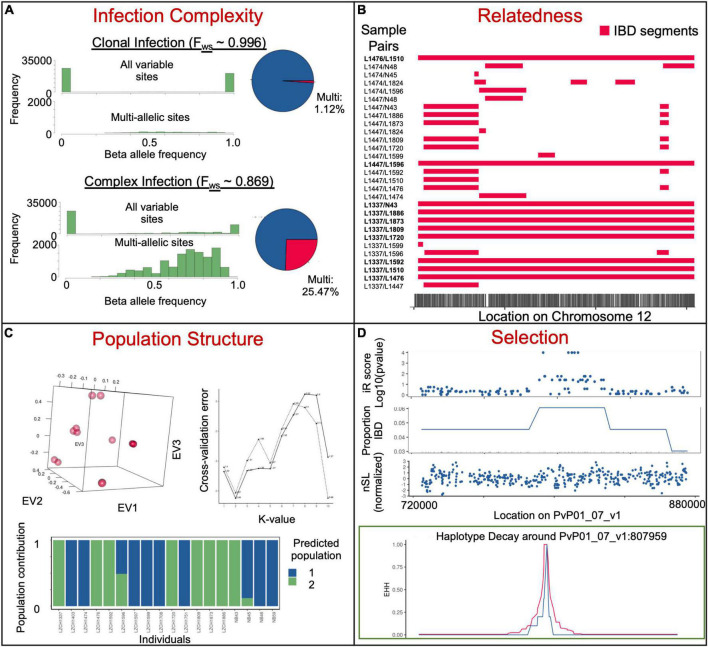
Example insights into parasite populations from whole-genome sequencing data. **(A)** Different beta allele frequencies for samples previously determined to be clonal (upper) and complex (lower) *via F*_ws_ analysis. The top histogram shows all sites, while the bottom shows only sites with reads mapping to more than one allele. Pie charts on the right represent the number of sites with more than 5% of mapped reads mapping to each of the two alleles. **(B)** Identical-by-descent segments on chromosome 12 within selected sample pairs. **(C)** Population structure of 18 clonal samples from the China–Myanmar border. PCA (top left), admixture *K*-value cross-validation identifying ideal population numbers based on SNPs (top right) and admixture analysis at the ideal *K*-value (bottom). **(D)** Genome scans for selection (iR score, proportion pairs IBD and nSL) on a fragment of chromosome 7 and haplotype decay 100 kb around an SNP at the center of a region based on 11 genetically distinct China-Myanmar border samples. Data is subset from a larger dataset (Brashear et al., 2020a).

Complexity of infection (COI) defines the degree of multiple infections and the similarity of those infections (Pearson et al., 2016). It is sometimes used to reflect the transmission intensity. However, the relationship between parasite prevalence and COI is often non-linear, likely sensitive to other factors, and in some cases, potentially more spurious in *P. vivax* than *P. falciparum* (Fola et al., 2017; Lopez and Koepfli, 2021). As genomics techniques have improved the ability to determine how closely related co-infections are, it also allows us to gleam the likelihood of a superinfection (from more than two separate mosquito bites) or co-transmission (more than two parasite clones from one mosquito bite) (Cowell et al., 2018). COI is frequently used to extrapolate the multiplicity of infection (MOI). Polyclonal samples are frequently omitted in population genomic methods because multiple infections can be problematic for certain profile-based analyses if separate genetic locations are called from two or more parasites. Therefore, inferring COI is typically one of the first analyses done in *Plasmodium* genomics studies. MOI may be estimated by genotyping at individual loci by amplicon deep sequencing, at a set of loci such as microsatellite markers or SNPs, or more recently, using WGS data (Zhong et al., 2018). When coverage is high enough for effective variant calling across a majority of the genome, WGS offers superior sensitivity in detecting multiclonal infections that are generally missed by genotyping a limited number of markers (Flannery et al., 2015; Ibrahim et al., 2020) and for differentiating closely-related clones such as the meiotic siblings in an infection (Figure 3A; Bright et al., 2014). Single-cell genomics provides the best resolution of the genetic differences of individual parasite clones within very complex infections (Nkhoma et al., 2020), but may be too costly for resolving infections when minor-allele clones are less than 5% or for assessing a large number of infections.

Multiple methods exist to estimate COI from whole-genome data. The within-host diversity (*F*_WS_) statistic is one popular method (Manske et al., 2012), although it is technically a measurement of inbreeding. *F*_WS_ compares within-sample, and within-population heterozygosity, and the resulting values are continuous in a range between 0 and 1, with higher values representing samples with higher inbreeding. While high levels of inbreeding are not necessarily due to a lack of clones, *F*_WS_ ≥ 0.95 is a popular cut-off for clonal infections. In addition to multiple implementations of *F*_WS_ (Chang et al., 2017), other methods for estimating the MOI have been developed for SNP data based on either minor allele frequency (MAF) distribution (Galinsky et al., 2015) or haplotype structure (Zhu et al., 2018). MAF-based methods include plotting or examining minor allele frequencies at each SNP to look for an unexpected number of heterozygous SNPs (Galinsky et al., 2015; Pearson et al., 2016; Brashear et al., 2019). This approach doesn’t require reference datasets which can be beneficial when looking at individual clones, but can also be biased by data with imperfect mapping. Haplotype structures identify stretches of the genome traveling together and look at the number of each haplotype to predict the number of genetically distinct parasites (Zhu et al., 2018). This can result in higher accuracy at delineating closely related parasites but predicting haplotypes from short-read data is unreliable without strong reference haplotypes. One method to mitigate this concern was employed by the EstMOI program, which looks at strings of SNPs on the same reads or read pairs (default 3 SNPs) as haplotypes and calculates parasite prevalence (Assefa et al., 2014). Importantly, amplification techniques may cause an overestimation or underestimation of minor clones within an infection. Methodological advances in haplotype prediction may bolster future population genomic analyses.

Relapses in *P. vivax* and *P. ovale* spp. increase the chance of multi-clonal infections and may result in high MOI even in low-endemicity settings. For example, amplicon sequencing of the *pvmsp1* gene detected over 90% of infections from low-endemicity areas of Cambodia as polyclonal; some infections contained as many as 10 parasite clones, and about half of the recurrent infections were attributable to relapses (Lin et al., 2015). Though the changes in MOI and malaria incidence are complex and their relationship is not linear, especially in species known for relapsing, reduced malaria transmission is generally associated with reduced MOI or increased levels of clonality. On a global level, eastern SE Asia and Oceania have more complex *P. vivax* infections than other parts of the globe, despite *P. falciparum* having the most complex infections within Africa (MalariaGEN et al., 2022). *F*_WS_ analysis of *P. vivax* populations showed Malaysia had a significantly higher proportion (84%) of monoclonal infection (*F*_WS_ ≥ 0.95) than other endemic areas such as Thailand, Indonesia, and Ethiopia (52–71%), consistent with the decline of vivax malaria incidence in Malaysia followed by unstable transmission and outbreaks (Auburn et al., 2018, 2019). Similarly, *P. vivax* infections from the China–Myanmar border also displayed a significantly higher level of clonality than in neighboring areas, reflecting lineage expansions during an outbreak (Brashear et al., 2020a).

## Characterizing populations with genome-wide single nucleotide polymorphisms

Knowledge about the genetic diversity and population structure of malaria parasites improves understanding of the epidemiology, transmission patterns, population dynamics, parasite distribution and movement, and response to control measures of natural parasite populations, thus providing appraisal and guidance for malaria control and elimination activities (Arnott et al., 2012). The genetic diversity of *P. vivax* populations has been extensively studied using single polymorphic genes (Sanger sequencing or amplicon deep sequencing), microsatellite markers, and more recently, SNPs (as a subset of loci, such as in barcodes, or genome-wide) (Auburn and Barry, 2017; Escalante and Pacheco, 2019). As NGS costs continue to decline, population genomic studies using genome-wide SNPs have gained popularity. Small-scale WGS of *P. vivax* field isolates started a decade ago (Chan et al., 2012), which culminated in large-scale genomic analyses of > 400 global *P. vivax* field isolates in 2016 (Hupalo et al., 2016; Pearson et al., 2016), and more recently > 1500 samples from across the globe (MalariaGEN et al., 2022). Together with other regional *P. vivax* sequencing efforts including Southeast Asia [Cambodia (Parobek et al., 2016), southern China (Shen et al., 2017), Malaysia (Auburn et al., 2019), Myanmar (Brashear et al., 2020a)], South America [Colombia (Winter et al., 2015), Peru (Flannery et al., 2015), and Brazil (De Oliveira et al., 2017, 2020)], and Africa [Ethiopia (Auburn et al., 2019; Ford et al., 2020)] (Figure 1 and Supplementary Table 1), *P. vivax* genomic studies delved into the demographic history of this parasite and detected events of parasite lineage expansion, contraction, or introduction. The use of genome-wide SNPs within core genomic regions, often exceeding 200,000, has dramatically increased the capability of population studies, facilitating the fine-grain mapping of population genetic structure, identification of genes under selection by host immunity or drugs, and resolution of the relationships of parasites within a population or even within a single host. Population differentiation can be gauged using grouping-agnostic methods such as principal component analysis (PCA), phylogeny, pairwise IBD, and admixture. It may also be more directly studied by comparing populations using Wright’s fixation index (*F*_ST_) and shared IBD (Figure 3). IBD is measured between each pair of individuals, while the average pairwise relatedness is population informative. *F*_ST_ is a population differentiation metric derived from allele frequencies within individual populations. Comparing shared IBD is more informative than *F*_ST_ for recent demographic events and datasets where parasites are closely related (Taylor et al., 2017).

Analysis of contemporary global *P. vivax* populations using genome-wide SNPs firmly established population differentiation among the continents and identified distinct populations from Oceania (PNG), South Asia (India), and Southeast Asia (Hupalo et al., 2016; MalariaGEN et al., 2022), with improved resolutions compared to microsatellites (Koepfli et al., 2015). Within continents, parasite populations generally conform to isolation by distance (MalariaGEN et al., 2022); populations from more distantly separated countries form distinctive clusters and correlate well to countries of origin, but parasites from neighboring countries are sometimes difficult to distinguish, even using genome-wide SNPs (De Oliveira et al., 2017; Brashear et al., 2020a). In South America, for example, population differentiation is evident between Mexico and Brazil but less clear between Peru and Colombia (Hupalo et al., 2016; De Oliveira et al., 2017). Similarly, parasites from Indonesia and Malaysia are well separated from those in the GMS (Auburn et al., 2018), but parasites from Cambodia and Vietnam are not readily differentiated, reflecting physical connectivity and frequent gene flow between the two countries (Pearson et al., 2016; Brashear et al., 2020a). Interestingly, however, the malaria-free central plain of Thailand serves as a gene flow barrier, resulting in substantial population division between parasites from the eastern and western GMS that can be distinguished with as few as four microsatellite markers (Kittichai et al., 2017). Population differences characterized by genomics can be harnessed to gauge and guide malaria control and elimination practice.

## Monitoring temporal population changes using genomics

Investigations of temporal changes of parasite populations offer a way to monitor the progress of malaria control and elimination. As elimination efforts increase selection pressure on parasites and reduce gene flow, parasite populations become more fragmented and structured, even though the effective population size may not vary significantly, as demonstrated in microsatellite data from parasites spanning a decade from the South Pacific and GMS (Waltmann et al., 2018; Li et al., 2020). WGS provides higher resolution to detect population contractions, bottlenecks, introductions, and expansions, all of which may occur during malaria elimination. For example, *P. vivax* subpopulations showing high degrees of IBD-sharing—suggestive of a history of severe bottleneck and/or clonal expansion—were identified in sites approaching malaria elimination, including Sabah, Malaysia (Auburn et al., 2018), and Panama (Buyon et al., 2020). Likewise, WGS of *P. vivax* parasites from a transmission hotspot in Brazil also detected a fragmented parasite structure, with nearly half of the samples sharing over 50% of their genome in IBD segments, indicating highly inbred parasite populations (De Oliveira et al., 2020). Interestingly, these sympatric lineages observed in Brazil and the predominant lineage identified in Panama are remarkably stable over time, suggesting the existence of persistent reservoirs (Buyon et al., 2020; De Oliveira et al., 2020). In addition to significant IBD sharing, population expansion or reduction may also be reflected in Tajima’s *D* values – e.g., a negative value may indicate population expansion (De Oliveira et al., 2017). Depending on the local scenarios, rapidly expanded parasite lineages may represent epidemic events such as outbreaks (Brashear et al., 2020a) or evolution adaptation of the parasites to selective pressures such as drug-resistant founder populations.

## Tracking parasite movement using genomics

The superb resolution of the parasite genotypes from WGS data makes the accurate tracking of the origins and movement of the parasites possible. With the ability to persist as liver hypnozoites, *P. vivax* and *P. ovale* spp. can be introduced as silent infections by migrant workers and travelers (Cao et al., 2016; Spanakos et al., 2018; Zhou et al., 2019). For non-endemic countries, genomic information can approximate where the parasites are imported from (Diez Benavente et al., 2020). In Panama, which is close to elimination, population genomics allowed accurate tracking of the origins of malaria parasites in patients with travel histories (Buyon et al., 2020). For endemic countries, malaria parasites between neighboring countries or different endemic “pockets” within a country have become increasingly divergent with the scale-up of malaria control activities. Thus, information about the sources and sinks of the parasites can be used to identify the routes of parasite movement and block parasite introduction (Buyon et al., 2020). With the shrinking of parasite populations and inbreeding within isolated populations, local parasites may become more closely related. Genetic divergence (e.g., estimated by pairwise *F*_ST_) or genetic relatedness (e.g., IBD-sharing) can be compared with geographical distances by the Mantel test to identify differentiation beyond what is expected from geographic isolation alone. In one instance, the identification of *P. vivax* parasites with significant IBD sharing from two sites 700 km apart in the Brazilian Amazon evidenced long-distance migration (De Oliveira et al., 2020), implying the presence of “corridors” for long-distance migration of parasites as suggested from microsatellite analysis of *P. vivax* parasites from Colombia (Pacheco et al., 2019). For geo-referenced parasite samples across a geographic region, the spatial patterns of gene flow (or migration routes) can be visually presented using Effective Estimation of Migration Surfaces (EEMS), a tool that maps relative migration rates among samples by comparing a matrix of pairwise sample dissimilarity to pairwise distance and identifying regions where dissimilarity is higher or lower than expected (Petkova et al., 2016). In the GMS, EEMS analysis of *P. falciparum* and *P. vivax* populations using genome-wide SNP data detected central Thailand and some international borders as common migration barriers for both species (Shetty et al., 2019; Brashear et al., 2020a), although the *P. vivax* samples were much more spatially sparse. It is noteworthy that both IBD correlations and EEMS assume equal transmission in both directions, requiring additional analysis to detect the migration sources. MIGRATE-N, which uses Bayesian inference to output posterior probability for proposed models, has been used with microsatellite data to infer the migration direction for *P. vivax* between China and Myanmar (Lo et al., 2017b), within Peru (Delgado-Ratto et al., 2016), and within Ethiopia (Lo et al., 2017a), but is not computationally feasible for the large number of SNPs frequently used in WGS studies. Fine-scaled mapping of asymmetrical migration to identify sources and sinks of migrating parasites, especially on small geographical scales and across political boundaries, may help make actionable plans to prevent parasite introduction during elimination.

## Detecting signatures of selection

Compared to *P. falciparum, P. vivax* shows much higher genetic diversity and genomic plasticity, which may enhance its ability to evade host immunity and develop resistance to chemotherapy (Neafsey et al., 2012; Hupalo et al., 2016; Pearson et al., 2016). Positive natural selection forces, such as antimalarial drugs, can lead to decreased genomic diversity at associated and linked sites, producing a “selection valley” of reduced haplotype heterozygosity. Accordingly, either a single advantageous genotype (hard sweep) or multiple adaptive genotypes (soft sweep) will rise to high frequency in the population. A good example is the hard transnational sweep of *P. falciparum* strains carrying PfK13 mutations and *plasmepsin 2/3* amplification in the GMS, resulting from dihydroartemisinin-piperaquine selection (Imwong et al., 2017). Studies highlighting drug selection as a more recent selective force shaping *P. falciparum* population genetic structure suggest that this principle should also apply to *P. vivax* (Miotto et al., 2015). Alternatively, diversifying selection may occur at antigenic sites where host immune responses discourage highly similar antigen sequences within a population. By looking at genome composition at the population level, researchers can understand the selective pressures driving parasite evolution and design more effective strategies guiding malaria elimination.

To detect signatures of natural selection, high-density SNPs from large-scale WGS data are necessary for high-transmission areas, where the level of recombination is high and pairwise linkage disequilibrium is low (Park et al., 2012). High linkage disequilibium or notable population structure may bias many statistics, as will insufficient sample sizes. Without phenotypic data, allele frequency-derived statistics such as nucleotide diversity (π), Tajima’s D, and the ratio of non-synonymous/synonymous mutation (π_*N*_/π_*S*_) may be used to identify genes under selection. This type of analysis allows the identification of genes showing elevated genetic diversity and positive D (balancing selection, e.g., surface proteins and antigens) or negative *D* values (negative selection or selection sweep) (Mu et al., 2007; Amambua-Ngwa et al., 2012; Pearson et al., 2016). The ability to gauge homozygosity over long chromosomal distances with the WGS data allows the applications of haplotype-based statistics such as integrative haplotype score (iHS), cross-population extended haplotype homozygosity (XP-EHH), number of segregating sites by length (nSL), and most recently the isorelate Xir to detect selective sweep within parasite populations. Within a population, an exceptionally long IBD region is likely to have undergone fewer recombination events, suggesting a deleterious effect of mutations in that region. With this in mind, IsoRelate creates the isorelate statistic (Xir) to determine the relatedness of individuals and find regions under selection. It was successfully used to detect drug resistance genes in *P. falciparum* (Henden et al., 2018). However, other statistics, such as iHS and XP-EHH, take advantage of a similar concept called “extended haplotype homozygosity,” which scans for especially long shared haplotypes. nSL differs only slightly from iHS in that it is capable of using segregating sites to estimate genetic distance rather than an explicit genetic map; a study using both nSL and iHS in *P. vivax* found overlaps in key selection signals (Parobek et al., 2016). XP-EHH and Rsb compare extended haplotypes between two populations and is useful when two populations of interest likely have different selective pressures. In multiple *P. vivax* populations, signatures of selection have been identified in invasion-related genes (e.g., *msp5*, *msp10*, and *ama1*), AP2 transcription factors, and genes potentially related to drug resistance (Parobek et al., 2016; Auburn et al., 2019; Table 1). As *P. vivax* populations from different geographic regions reflect different demographic histories, such as adaptation to local hosts and vectors and different drug histories, their comparison may reveal population-specific selection pressure (Hupalo et al., 2016). The divergence between New World and Old World *P. vivax* populations on the *Pvs47* gene, the ortholog of which in *P. falciparum* has been selected by the New World vectors, may likewise be due to selection by different vectors (Hupalo et al., 2016).

**TABLE 1:** Genes implicated as being under directional selection from population genomics studies.

Gene ID*	Description	Mutations	WGS-based evidence of selection	References
PVP01_0526600	dhfr	57L; 58R; 61M; 117N	iHS, XP-EHH, *F*_ST_, Rsb, CNV	Flannery et al., 2015; Hupalo et al., 2016; Pearson et al., 2016; Diez Benavente et al., 2017; Auburn et al., 2019; Brashear et al., 2020a
PVP01_1429500	dhps	383G; 553G	XP-EHH; iHS; *F*_ST_; Linkage Disequilibrium; Rsb; Low Diversity	Winter et al., 2015; Hupalo et al., 2016; Pearson et al., 2016; Auburn et al., 2019; Brashear et al., 2020a
PVP01_1010900	mdr1	Y976F; F1076L	iHS, Rsb; nSL; CNV; XP-EHH; Fst; Diversity	Auburn et al., 2016b; Hupalo et al., 2016; Parobek et al., 2016; Pearson et al., 2016; Auburn et al., 2019
PVP01_0203000	mrp1	T234M; T259I; T259R; Q906E; L1207I; Y1393D; V1478D	iHS; Rsb; nSL; XP-EHH; CNV	Flannery et al., 2015; Parobek et al., 2016; Pearson et al., 2016; Diez Benavente et al., 2017; Auburn et al., 2019; Brashear et al., 2020a
PVP01_1317400	VDAC	NA	iHS; Rsb; XP-EHH	Pearson et al., 2016; Auburn et al., 2019
PVP01_1418100	AP2-G3	NA	nSL; iHS; XP-EHH	Parobek et al., 2016; Auburn et al., 2019; Brashear et al., 2020a
PVP01_1468200	Hypothetical protein	NA	CNV; iHS, XP-EHH	Pearson et al., 2016
PVP01_0109300	crt	NA	Rsb	Auburn et al., 2019
PVP01_0623800	dbp	NA	CNV	Parobek et al., 2016; Auburn et al., 2019
PVP01_1439300	SET10	NA	nSL; iHS	Parobek et al., 2016; Diez Benavente et al., 2017
PVP01_0417200	SERA5	NA	nSL; iHS	Parobek et al., 2016; Diez Benavente et al., 2017
PVP01_0940100	AP2-G5	NA	nSL	Parobek et al., 2016; Brashear et al., 2020a
PVP01_0922500	PDK-1	NA	*F*_ST_; iHS	Hupalo et al., 2016; Diez Benavente et al., 2017
PVP01_0709800	CRMP1	NA	*F*_ST_; iHS	Hupalo et al., 2016; Diez Benavente et al., 2017
PVP01_1208000	Pvs47	NA	*F*_ST_; Rsb	Hupalo et al., 2016; Diez Benavente et al., 2017
PVP01_1447300	MRP2	NA	nSL; XP-EHH	Brashear et al., 2020a
PVP01_1453300	VP2	NA	nSL; XP-EHH	Brashear et al., 2020a
PVP01_1124800	PIGL	NA	nSL; XP-EHH	Brashear et al., 2020a
PVP01_1334400	ABCk2	NA	nSL; XP-EHH	Brashear et al., 2020a
PVP01_1460600	SNF7	NA	nSL; XP-EHH	Brashear et al., 2020a
PVP01_0418400	MSP5	NA	iHS; Rsb	Diez Benavente et al., 2017
PVP01_0735100	Exported protein	NA	iHS; Rsb	Diez Benavente et al., 2017
PVP01_0803900	WD repeat protein	NA	iHS; Rsb	Diez Benavente et al., 2017

*Gene ID is according to the P01_v1 annotation. ABCk2, ABC-1 family atypical protein kinase; CDPK1, 3-phosphoinositide dependent protein kinase-1; CRMP1, cysteine repeat modular protein 1; crt, chloroquine resistance transporter; dbp, duffy-binding protein; dhfr, dihydrofolate reductase; dhps, dihydropteroate synthetase; mdr1, multidrug resistance protein 1; mrp1, multidrug resistance-associated protein 1; mrp2, multidrug resistance-associated protein 2; MSP5, merozoite surface protein 5; PIGL, N-acetylglucosaminyl-phosphatidylinositol de-N-acetylase; SERA5, serine-repeat antigen 5; SET10, histone-lysine N-methyltransferase 10; SNF7, vacuolar-sorting protein SNF7; VDAC, voltage-dependent anion-selective channel protein; VP2, vacuolar-type H^+^ pumping pyrophosphatase.

Selection signals from population genomics are especially useful for neglected malaria parasites where resources are scarce and traditional means of genetics are difficult. Genetic linkage mapping using experimental crosses and genome-wide association studies (GWAS) have successfully mapped loci associated with drug resistance in *P. falciparum* (Su et al., 2007; Volkman et al., 2017). To date, no GWAS has been carried out for the neglected parasites, although drug resistance phenotypes can be obtained from clinical studies or *ex vivo* drug assays. Clinical phenotypic data are typically more variable in nature, necessitating larger sample sizes to find genuine associations. *Ex vivo* assays are more controllable but labor intensive, particularly for samples that are not easily cultured. Sá et al. (2019) recently overcame tremendous barriers to perform a genetic cross of two subpopulations of an NIH-1993 line of *P. vivax* in a splenectomized chimpanzee. By comparing the prevalence of 37 polymorphic markers from chloroquine (CQ)-resistant and –sensitive parental strains before and after CQ administration, the authors calculated an effect e-value for each marker and identified those on chromosome 1 with consistently high e-values across progeny pairs. Targeted sequencing on chromosome 1 and transcriptomic analysis suggested that CQ resistance may be mediated by changes in *pvcrt* transcription resulting from an upstream motif. While sample sizes for these analyses were small (4–10 linkage group selection pairs), and it is uncertain how mutations in primate-adapted strains may affect the results, they represent an important step forward in phenotypic analyses in *P. vivax*. In general, however, the lack of a long-term culture system and the need for non-human primates make it less feasible to apply phenotypic assays to genetic mapping in neglected malaria parasites. Therefore, we rely heavily on population genomics to infer underlying resistance mechanisms.

Signatures of selection between populations may reflect different drug histories and provide hints about the mechanisms of drug resistance in this parasite. Strong selective sweeps have been detected in genomic regions harboring *Pvdhps* and *Pvdhfr*, mediating resistance to the antifolate drugs sulfadoxine and pyrimethamine (SP), respectively (Winter et al., 2015; Hupalo et al., 2016; Parobek et al., 2016; Pearson et al., 2016; Brashear et al., 2020a). These genes also show significant geographic divergence between the New World and Old World, between PNG and Thailand, and even between geographically adjacent countries, reflecting the varied histories of SP as a treatment for sympatric *P. falciparum* (Hupalo et al., 2016; Pearson et al., 2016; Earland et al., 2019; Brashear et al., 2020a). Of note, some of the mutations in PvDHFR and PvDHPS are relatively rare in South and Central American *P. vivax* populations (De Oliveira et al., 2017). CQ resistance (CQR) has been detected in *P. vivax* populations from many endemic regions, but the resistance mechanism is not understood and appears different from *P. falciparum* (Price et al., 2014). Unlike the selective sweep found at the *pfcrt* gene, the major determinant of CQR in *P. falciparum*, no selection signals were identified at the *pvcrt* locus (Parobek et al., 2016; Pearson et al., 2016) except an extended haplotype upstream of *pvcrt* differing between Thai and Ethiopian *P. vivax* populations (Auburn et al., 2019). This corroborates findings from a genetic cross showing that *pvcrt* expression levels in the CQ-sensitive and -resistant parasites were correlated with variations in the *pvcrt* upstream sequence motifs, potential sites for transcription factors (Sá et al., 2019). In this context, positive selection in AP2-domain transcription factors may suggest their involvement in regulating the expression of drug resistance genes (Dharia et al., 2010; Parobek et al., 2016; Auburn et al., 2019). *Pvmdr1*, which emerged as a potential CQR marker during *ex vivo* studies (Suwanarusk et al., 2007), has displayed substantial population-level variation at major mutations (Y976F and F1076L). Both mutations were fixed in Indonesia, where high-grade CQR is found, with low to modest prevalence in the GMS (Auburn et al., 2019; Brashear et al., 2020a). In *P. vivax* from Indonesia, a strong selection signal was also identified downstream of, but not overlapping with, *pvmdr1* (Pearson et al., 2016). In addition, population genomic studies of global *P. vivax* populations have identified signals of selection in other chromosomal regions, including around genes encoding putative transporters such as multidrug resistance-associated protein 1 (*pvmrp1*) and 2 (*pvmrp2*) (Dharia et al., 2010; Flannery et al., 2015; Parobek et al., 2016; Pearson et al., 2016; Auburn et al., 2019; Brashear et al., 2020a).

Copy number variations (CNVs) may also differ among populations and potentially lead to drug resistance. CNVs with a marked geographical difference in frequency were identified between *P. vivax* populations from Oceania and the GMS, some of which harbor genes related to drug resistance (Pearson et al., 2016). Among them, *pvmdr1* amplification, previously associated with mefloquine resistance (Suwanarusk et al., 2008), had ∼20% prevalence in Thailand (which has extensively deployed mefloquine to treat *P. falciparum*) but was absent in Indonesia or Ethiopia (Auburn et al., 2016b,^2019^; Pearson et al., 2016). A short 3-kb duplication on chromosome 14, including the gene *PVX_101445*, was found only in Oceania parasites (Pearson et al., 2016). Detecting CNVs from short-read data can be tricky due to mismapping, especially if reference genomes are not complete, while methods incorporating discordant reads and/or read depth have found some success. Genome amplification techniques prior to sequencing (e.g., SWGA) can worsen this technical concern, while long-read sequencing data is considered more reliable for CNV detection.

Population genomic studies have identified various genes under selection, some of which may mediate drug resistance, but the lack of a long-term *P. vivax* culture or convenient animal models for genetic manipulation precludes direct evaluations of their roles in drug resistance. The expression of these identified *P. vivax* genes in transgenic *P. knowlesi* (Verzier et al., 2019), a closely related species that can be cultured in human RBCs, and adaptation of the marker-free CRISPR gene-editing technology in *P. knowlesi* (Mohring et al., 2020), have opened a new way for downstream functional analysis of *P. vivax* genes. Incorporating population genomics into the study of neglected malaria parasites may provide insights into the nature of the selective pressure.

## Using single nucleotide polymorphism barcodes for high-throughput genotyping

Even with the continuous reduction of WGS costs, it is still prohibitively expensive to perform large-scale population genomic studies of malaria parasites. Yet, the availability of WGS data of parasites from various endemic areas such as those generated from the MalariaGEN Consortium has informed researchers to design ‘genetic barcodes’, which offer a reductionist approach to inferring transmission networks and population structures by using a small set of SNPs (Daniels et al., 2008; Preston et al., 2014; Baniecki et al., 2015). Barcode SNPs are selected as highly differentiated among subpopulations and can be affordably genotyped from small amounts of blood such as dried blood spots using various platforms such as the high resolution melting analysis (Baniecki et al., 2015), molecular inversion probes (Verity et al., 2020), or the Sequenom platform (Omedo et al., 2017). The *P. falciparum* barcode consisting of 24 SNPs (Daniels et al., 2008; Preston et al., 2014) has proved to be applicable to studying parasite populations in a range of endemic settings of the world (Auburn and Barry, 2017), although a much larger SNP set may be needed for the resolution of global parasite populations and IBD analysis. The barcodes for *P. vivax* populations appear to require more SNPs, likely due to higher genetic diversity (Neafsey et al., 2012). A set of 42 SNPs was initially designed based on a limited number of *P. vivax* genomes available at that time (Baniecki et al., 2015). Though the 42-SNP barcode can discriminate parasites from different continents, it was insufficient to differentiate parasite populations from South and Central America or countries of Southeast Asia (De Oliveira et al., 2017; Brashear et al., 2020a). The addition of 37 out of 42 SNPs from the initial barcode to a set of 28 sites selected with machine learning improved its resolution for country-level classification with more flexibility for missing data (Trimarsanto et al., 2019). Recently, as *P. vivax* WGS data have become available from more geographic regions, a new 71-SNP barcode has been designed, allowing the geographic origins of parasites to be predicted at 91.4% accuracy (Diez Benavente et al., 2020). Though this new barcode remains to be tested, because barcodes are designed for specific purposes based on variability expected in that dataset, barcodes produced from global datasets often underperform in local and regional analyses, prompting additional barcodes for more targeted use in tracking transmission within countries or between neighboring countries. For example, barcodes based on 100 SNPs and 36 SNPs were shown to be able to differentiate parasites in South and Central Americas and the GMS, respectively (De Oliveira et al., 2017; Brashear et al., 2020a), while their performance remains to be evaluated. Establishing the origin of samples is important when determining malaria-free status by the WHO (World Health Organization [WHO], 2021), but it is important to consider the purpose and scope of a barcode in use.

## Implications for zoonotic human-infecting malaria parasites

Zoonotic malaria species infecting humans such as *P. knowlesi, P. cynomolgi*, and *P. simium* are increasingly recognized as additional targets for malaria elimination. Malaysia has been cleared of human malaria since 2018 but continues to report thousands of human cases of *P. knowlesi* (World Health Organization [WHO], 2021). As global deforestation continues, the displacement of primate species that carry zoonotic malaria may further increase the risk of transmission to humans (Stark et al., 2019). Genomic studies of the zoonotic species are also challenging, and the parasites infecting humans may be genetically divergent from those infecting their natural non-human primate hosts (Assefa et al., 2015). The adaptation of zoonotic species to *in vitro* culture in human RBCs has opened new venues to study these parasite species in the lab, which may serve as model systems for functional genomics of their closely related human malaria species, such as *P. vivax* (Moon et al., 2013; Chua et al., 2019). In 2008, *P. knowlesi* from a rhesus macaque was sequenced, revealing a 23.5 Mb nuclear genome (Pain et al., 2008). Subsequent long-read sequencing on the PacBio platform with hi-C correction improved the annotation of the *P. knowlesi* genome of 24.77 Mb to consist of 14 contigs with 25 gaps (Lapp et al., 2018). Recently, the Oxford nanopore long-read sequencing has opened the door to *de novo* assemblies of clinical field samples (Oresegun et al., 2022). *P. cynomolgi* was sequenced in 2012, resulting in three draft genomes (Tachibana et al., 2012); the assembly was later improved to have 56 contigs with no gaps, accounting for 6632 genes (Pasini et al., 2017). The *P. simium* draft genome was only recently sequenced, containing over 2000 scaffolds (Mourier et al., 2021). Improvement in the quality and annotation of these reference genomes will benefit future functional and population genomic studies.

Population genomics techniques have a clear benefit in neglected *Plasmodium* species due to the ability to amass a large amount of data with few samples and little *a priori* knowledge. Of the zoonotic species, population genomics studies have been undertaken primarily on *P. knowlesi* due to the large number of human infections occurring in Malaysia (Divis et al., 2021). Most notably, these studies have provided clear evidence that *P. knowlesi* populations are structured not just geographically and between clinical and laboratory samples but also by primate hosts (Assefa et al., 2015; Hocking et al., 2020). The original division between two known monkey hosts—the pig-tailed macaque and the long-tailed macaque—was somewhat intuitive (Assefa et al., 2015). A recent finding that peninsular samples actually had three genomic subpopulations in overlapping geographic regions (Hocking et al., 2020) leaves the possibility that there may be additional host reservoirs or additional factors contributing to parasite transmission. High transmission intensity in monkey hosts leads to high genomic and within-host diversity (Lee et al., 2011; Assefa et al., 2015). In *P. simium*, advancement in population genomics includes compelling evidence that the species diverged from *P. vivax* in a rare occurrence of reverse zoonosis (De Oliveira et al., 2021; Mourier et al., 2021). Analysis of 11 *P. simium* samples revealed high genomic similarity to new-world *P. vivax* samples, but not to old-world *P. vivax* samples (De Oliveira et al., 2021), supporting the hypothesis that *P. vivax* spread to the Americas and diverged from its Asian cousins before adapting to local macaques. Finally, very little genomic research has been done with *P. cynomolgi* field populations. The development of genomic tools and resources for studying zoonotic malaria species may provide crucial findings on the parasite’s host adaptation and the biology of closely related human parasites.

## Conclusion

With technical improvements to enrich parasite genomes from very small amounts of DNA, the number of genomes of the neglected malaria parasites sequenced is rapidly growing, providing a new means to address many aspects of parasite biology. High-coverage WGS paves the way to precisely determine the relatedness of recurring parasites with parasites from the initial infection, setting up a platform for studying the biology of relapses (Cowell et al., 2018; Popovici et al., 2018). Population genomics provides a more accurate way to infer the connectivity and ancestry of the parasite populations. Under the malaria elimination scenario, analysis of longitudinal samples enables the detection of population events such as contraction, bottleneck, and expansion, allowing close monitoring of the malaria elimination progress (Auburn et al., 2018; Buyon et al., 2020). The availability of parasite genomes from many endemic regions globally and the genomic resolution of parasite populations allowed the conclusive detection of parasite introduction (Buyon et al., 2020), which is critical for areas approaching and achieving elimination status. Finally, identifying genomic regions and genes under selection provides drug resistance candidate markers for future evaluation (Pearson et al., 2016).

Population genomics of neglected malaria parasites offers a new frontier of malaria research. While the malaria community has recently generated an impressive number of over 2200 whole-genome sequences for *P. vivax* (Supplementary Table 1), the studies on other neglected malaria parasites have just begun (Ibrahim et al., 2020). In *P. ovale* spp. and *P. malariae*, researchers would benefit immensely from large numbers of genomic data collected from various populations to hasten our understanding of their epidemiology and biology. Insights into how they transmit and recur or relapse in different settings will become especially important as *P. falciparum* decreases and other species stand in the way of malaria elimination. In simian species, including *P. knowlesi, P. cynomolgi*, and *P. simium*, population genomics may give insights into their transmission and possible additional hosts, and may even help researchers assess the threat of human transmission. For *P. vivax*, increased sample acquisition on finer geographical scales remains an important objective, especially for tracking regional parasite movement. We still lack *P. vivax* genomes from west Africa, where Duffy negativity is most prevalent. Additional locations where there are relatively large numbers of cases but relatively few WGS samples include the areas between Bangladesh and Myanmar, and northeastern South America, specifically around Venezuela (Figure 1 and Supplementary Table 1). Furthermore, with technological breakthroughs in WGS, better genome assemblies from long-read sequencing may be realized soon (Rutledge et al., 2017a). Improved reference genomes will improve population genomic studies in neglected malaria parasites, serving haplotype reference panels for phasing inference and genetic maps for models reliant on genetic distance. We envision that the increasing popularity of population genomics of neglected malaria parasites will translate into a genomic surveillance practice in malaria elimination.

## Author contributions

AB and LC conceived, drafted, revised, and gave final approval to the article. Both authors contributed to the article and approved the submitted version.

## Supplementary material

Supplementary Table 1 Whole-genome sequences available for *P. vivax* grouped by study and location.
