# Supplementary material for: Population genomics in neglected malaria parasites
Source: Front Microbiol. 2022 Sep 8;13:984394. doi: 10.3389/fmicb.2022.984394 (PMC9493318; doi:10.3389/fmicb.2022.984394)
Supplement: Supplementary Table 1 — Whole-genome sequences available for P. vivax grouped by study and location. [file Table_1.PDF]

Table 1. Whole-genome sequences available for *P . vivax* grouped by study and location

Study	Data Source	Country	Location Description	WGS Samples described
Malariagen4;1157-PV-MULTI-PRICE	ENA PRJNA240356-PRJNA240533; PRJNA295233	Afghanistan	Jalalabad	155
Malariagen4;1157-PV-MULTI-PRICE	ENA PRJNA240356-PRJNA240533; PRJNA295233	Afghanistan	Laghman	95
Benavante et al. 2021	ENA PRJEB44419	Afghanistan	NA	26
Malariagen4;1157-PV-MULTI-PRICE	ENA PRJNA240356-PRJNA240533; PRJNA295233	Bangladesh	Bangladesh	28
Benavante et al. 2021	ENA PRJEB44419	Bangladesh	NA	1
Malariagen4;1157-PV-MULTI-PRICE	ENA PRJNA240356-PRJNA240533; PRJNA295233	Bhutan	Bhutan	9
Hupalo et al. 2016	NCBI PRJNA240356–PRJNA240533	Brazil	Acrelandia, Acre	7
Malariagen4;1046-PV-BR-FERRERIA	ENA PRJNA240356-PRJNA240533; PRJNA295233	Brazil	Brazil	5
Malariagen4;1128-PV-MULTI-GSK	ENA PRJNA240356-PRJNA240533; PRJNA295233	Brazil	Manaus	37
de Oliveira et al. 2020	NCBI PRJNA643698	Brazil	Mancio Lima	38
Pearson et al. 2016	ENA PRJEB2140	Brazil	NA	3
de Oliveira et al. 2017	NCBI PRJNA350554	Brazil	Northwest (Remansinho & Acrelandia)	9
Hupalo et al. 2016	NCBI PRJNA240356–PRJNA240533	Brazil	Plácido de Castro, Acre	13
Malariagen4;1128-PV-MULTI-GSK	ENA PRJNA240356-PRJNA240533; PRJNA295233	Brazil	Porto Velho	7
Parobek et al. 2016	NCBI PRJNA295233	Cambodia	Battambang	9
Parobek et al. 2016	NCBI PRJNA295233	Cambodia	Kampot	9
Chan et al. 2012	NCBI PRJNA175266	Cambodia	NA	3
Pearson et al. 2016	ENA PRJEB2140	Cambodia	NA	40
Malariagen4;1128-PV-MULTI-GSK	ENA PRJNA240356-PRJNA240533; PRJNA295233	Cambodia	Oddar Meanchey	73
Parobek et al. 2016	NCBI PRJNA295233	Cambodia	Oddar Meanchey	60
Malariagen4;1044-PF-KH-FAIRHURST	ENA PRJNA240356-PRJNA240533; PRJNA295233	Cambodia	Pursat	79
Malariagen4;1044-PF-KH-FAIRHURST	ENA PRJNA240356-PRJNA240533; PRJNA295233	Cambodia	Ratanakiri	3
Popvici et al. 2018	NCBI PRJNA420510	Cambodia	Ratanakiri	20
Malariagen4;1157-PV-MULTI-PRICE	ENA PRJNA240356-PRJNA240533; PRJNA295233	China	Anhui	5
Shen et al. 2017	NCBI PRJNA284437	China	CMB; Tengchong	6
Pearson et al. 2016	ENA PRJEB2140	China	NA	1
Malariagen4;1157-PV-MULTI-PRICE	ENA PRJNA240356-PRJNA240533; PRJNA295233	Colombia	Antioquia	8
Malariagen4;1157-PV-MULTI-PRICE	ENA PRJNA240356-PRJNA240533; PRJNA295233	Colombia	Bolivar	1
Hupalo et al. 2016	NCBI PRJNA240356–PRJNA240533	Colombia	Buenaventura, Valle del Cauca	3
Malariagen4;1128-PV-MULTI-GSK	ENA PRJNA240356-PRJNA240533; PRJNA295233	Colombia	Cali	5
Malariagen4;1157-PV-MULTI-PRICE	ENA PRJNA240356-PRJNA240533; PRJNA295233	Colombia	Choco	23

Malariagen4;1157-PV-MULTI-PRICE	ENA PRJNA240356-PRJNA240533; PRJNA295233	Colombia	Colombia	3
Malariagen4;1157-PV-MULTI-PRICE	ENA PRJNA240356-PRJNA240533; PRJNA295233	Colombia	Cordoba	3
Malariagen4;1157-PV-MULTI-PRICE	ENA PRJNA240356-PRJNA240533; PRJNA295233	Colombia	Córdoba	1
Winter et al. 2015	NCBI PRJNA285409	Colombia	Cordóba	8
Malariagen4;1157-PV-MULTI-PRICE	ENA PRJNA240356-PRJNA240533; PRJNA295233	Colombia	Pichimá	1
Hupalo et al. 2016	NCBI PRJNA240356–PRJNA240533	Colombia	Quibdó, Choco	3
Malariagen4;1157-PV-MULTI-PRICE	ENA PRJNA240356-PRJNA240533; PRJNA295233	Colombia	Santa Cecilia	16
Malariagen4;1157-PV-MULTI-PRICE	ENA PRJNA240356-PRJNA240533; PRJNA295233	Colombia	Tierralta	20
Hupalo et al. 2016	NCBI PRJNA240356–PRJNA240533	Colombia	Tierralta, Cordoba	23
Hupalo et al. 2016	NCBI PRJNA240356–PRJNA240533	Colombia	Tumaco, Narino	2
Malariagen4;1157-PV-MULTI-PRICE	ENA PRJNA240356-PRJNA240533; PRJNA295233	El Salvador	El Salvador	1
Benavante et al. 2021	ENA PRJEB44419	Eritrea	NA	11
Malariagen4;1098-PF-ET-GOLASSA	ENA PRJNA240356-PRJNA240533; PRJNA295233	Ethiopia	Amhara	19
Auburn et al. 2019	ENA PRJEB4409	Ethiopia	Arbaminch	8
Auburn et al. 2019	ENA PRJEB4409	Ethiopia	Badowacho	6
Malariagen4;1157-PV-MULTI-PRICE	ENA PRJNA240356-PRJNA240533; PRJNA295233	Ethiopia	Batu	3
Malariagen4;1157-PV-MULTI-PRICE	ENA PRJNA240356-PRJNA240533; PRJNA295233	Ethiopia	Bishoftu	4
Malariagen4;1128-PV-MULTI-GSK	ENA PRJNA240356-PRJNA240533; PRJNA295233	Ethiopia	Gondar	28
Auburn et al. 2019	ENA PRJEB4409	Ethiopia	Halaba	3
Auburn et al. 2019	ENA PRJEB4409	Ethiopia	Hawassa	7
Lo et al. 2019	ENA PRJEB10888	Ethiopia	Jimma	24*
Malariagen4;1128-PV-MULTI-GSK	ENA PRJNA240356-PRJNA240533; PRJNA295233	Ethiopia	Jimma	20
Ford et al. 2020	ENA PRJEB10888	Ethiopia	Jimma, Southwestern Ethiopia	20
Benavante et al. 2021	ENA PRJEB44419	Ethiopia	NA	6
Malariagen4;1098-PF-ET-GOLASSA	ENA PRJNA240356-PRJNA240533; PRJNA295233	Ethiopia	Oromia	69
Malariagen4;1157-PV-MULTI-PRICE	ENA PRJNA240356-PRJNA240533; PRJNA295233	Ethiopia	South Nations Nationalities and Peoples' Region	36
Benavante et al. 2021	ENA PRJEB44419	Guyana	NA	3
Hupalo et al. 2016	NCBI PRJNA240356–PRJNA240533	India	Chennai, Tamil Nadu	8
Malariagen4;1157-PV-MULTI-PRICE	ENA PRJNA240356-PRJNA240533; PRJNA295233	India	India (returning traveller)	1
Malariagen4;1157-PV-MULTI-PRICE	ENA PRJNA240356-PRJNA240533; PRJNA295233	India	Indore (returning traveller)	1
Malariagen4;1157-PV-MULTI-PRICE	ENA PRJNA240356-PRJNA240533; PRJNA295233	India	Mumbai (returning traveller)	2
Benavante et al. 2021	ENA PRJEB44419	India	NA	38
Hupalo et al. 2016	NCBI PRJNA240356–PRJNA240533	India	NA	1

Pearson et al. 2016	ENA PRJEB2140	India	NA	1
Pearson et al. 2016	ENA PRJEB2140	Indonesia	NA	55
Malariagen4;1154-PV-TH-PRICE	ENA PRJNA240356-PRJNA240533; PRJNA295233	Indonesia	Papua Indonesia	253
Malariagen4;1157-PV-MULTI-PRICE	ENA PRJNA240356-PRJNA240533; PRJNA295233	Indonesia	Papua Indonesia (returning traveller)	29
Malariagen4;1157-PV-MULTI-PRICE	ENA PRJNA240356-PRJNA240533; PRJNA295233	Iran	Iran	15
Pearson et al. 2016	ENA PRJEB2140	Laos	NA	2
Malariagen4;1102-PF-MG-RANDRIANARIVELOJOSIA	ENA PRJNA240356-PRJNA240533; PRJNA295233	Madagascar	Maevatanana	1
Chan et al. 2012	NCBI PRJNA175266	Madagascar	NA	2
Chan et al. 2013	NCBI PRJNA175266	Madagascar	NA	1
Pearson et al. 2016	ENA PRJEB2140	Madagascar	NA	1
Malariagen4;1157-PV-MULTI-PRICE	ENA PRJNA240356-PRJNA240533; PRJNA295233	Malaysia	Klang	1
Pearson et al. 2016	ENA PRJEB2140	Malaysia	NA	6
Auburn et al. 2018	NCBI PRJEB2140	Malaysia	Sabah	51
Malariagen4;1157-PV-MULTI-PRICE	ENA PRJNA240356-PRJNA240533; PRJNA295233	Malaysia	Sabah	108
Hupalo et al. 2016	NCBI PRJNA240356–PRJNA240533	Mexico	Carrillo, Chiapas	1
Hupalo et al. 2016	NCBI PRJNA240356–PRJNA240533	Mexico	Frontera Hidalgo, Chiapas	1
Hupalo et al. 2016	NCBI PRJNA240356–PRJNA240533	Mexico	Huehuetán, Chiapas	1
Hupalo et al. 2016	NCBI PRJNA240356–PRJNA240533	Mexico	Tapachula, Chiapas	16
Hupalo et al. 2016	NCBI PRJNA240356–PRJNA240533	Mexico	Tuxtla Chico, Chiapas	1
Malariagen4;1052-PF-TRAC-WHITE	ENA PRJNA240356-PRJNA240533; PRJNA295233	Myanmar	Bago	1
Brashear et. al 2020	NCBI PRJNA603279	Myanmar	CMB; Laiza/Nanbang	23
Hupalo et al. 2016	NCBI PRJNA240356–PRJNA240533	Myanmar	Laiza township, Kachin State	8
Pearson et al. 2016	ENA PRJEB2140	Myanmar	NA	1
Benavante et al. 2021	ENA PRJEB44419	Pakistan	NA	35
Hupalo et al. 2016	NCBI PRJNA240356–PRJNA240533	Papua New Guinea	Alexishafen, Madang Province	7
Malariagen4;1050-PV-PN-MUELLER	ENA PRJNA240356-PRJNA240533; PRJNA295233	Papua New Guinea	East Sepik	6
Malariagen4;1050-PV-PN-MUELLER	ENA PRJNA240356-PRJNA240533; PRJNA295233	Papua New Guinea	Madang	14
Hupalo et al. 2016	NCBI PRJNA240356–PRJNA240533	Papua New Guinea	Modillon	1
Pearson et al. 2016	ENA PRJEB2140	Papua New Guinea	NA	11
Malariagen4;1157-PV-MULTI-PRICE	ENA PRJNA240356-PRJNA240533; PRJNA295233	Papua New Guinea	Papua New Guinea (returning traveller)	3
Hupalo et al. 2016	NCBI PRJNA240356–PRJNA240533	Papua New Guinea	Yagaum	8
Hupalo et al. 2016	NCBI PRJNA240356–PRJNA240533	Peru	Delta 1, Madre de Dios, South Amazon	6
Cowell et al. 2015	NCBI SRP095853	Peru	Iquitos	18

Cowell et al. 2018	NCBI SRP132126	Peru	Iquitos	51
Malariagen4;1128-PV-MULTI-GSK	ENA PRJNA240356-PRJNA240533; PRJNA295233	Peru	Iquitos	76
Hupalo et al. 2016	NCBI PRJNA240356–PRJNA240533	Peru	Iquitos, Loreto, North Amazon	2
Flannery et al. 2015	NCBI PRJNA272364	Peru	Madre de Dios	10
Hupalo et al. 2016	NCBI PRJNA240356–PRJNA240533	Peru	Mazán, Loreto, North Amazon	10
Hupalo et al. 2016	NCBI PRJNA240356–PRJNA240533	Peru	Padrecocha, Loreto, North Amazon	11
Hupalo et al. 2016	NCBI PRJNA240356–PRJNA240533	Peru	Puerto America, Loreto, North Amazon	4
Hupalo et al. 2016	NCBI PRJNA240356–PRJNA240533	Peru	Santo Tomás, Loreto, North Amazon	10
Hupalo et al. 2016	NCBI PRJNA240356–PRJNA240533	Peru	Sullana, Piura, North Coast	4
Benavante et al. 2021	ENA PRJEB44419	Philippines	NA	1
Malariagen4;1128-PV-MULTI-GSK	ENA PRJNA240356-PRJNA240533; PRJNA295233	Philippines	Rio Tuba	6
Malariagen4;1047-PV-LK-KARUNAWEERA	ENA PRJNA240356-PRJNA240533; PRJNA295233	Sri Lanka	Kataragama	2
Pearson et al. 2016	ENA PRJEB2140	Sri Lanka	NA	1
Malariagen4;1157-PV-MULTI-PRICE	ENA PRJNA240356-PRJNA240533; PRJNA295233	Sudan	Kassala	13
Benavante et al. 2021	ENA PRJEB44419	Sudan	NA	7
Malariagen4;1128-PV-MULTI-GSK	ENA PRJNA240356-PRJNA240533; PRJNA295233	Thailand	Bangkok	48
Hupalo et al. 2016	NCBI PRJNA240356–PRJNA240533	Thailand	Kanchanaburi	20
Malariagen4;1128-PV-MULTI-GSK	ENA PRJNA240356-PRJNA240533; PRJNA295233	Thailand	Mae Sot	4
Pearson et al. 2016	ENA PRJEB2140	Thailand	NA	92
Malariagen4;1052-PF-TRAC-WHITE	ENA PRJNA240356-PRJNA240533; PRJNA295233	Thailand	Sisaket	2
Malariagen4;1154-PV-TH-PRICE	ENA PRJNA240356-PRJNA240533; PRJNA295233	Thailand	Tak	42
Malariagen4;1128-PV-MULTI-GSK	ENA PRJNA240356-PRJNA240533; PRJNA295233	Thailand	Umphang	11
Malariagen4;1154-PV-TH-PRICE	ENA PRJNA240356-PRJNA240533; PRJNA295233	Thailand	Wangpha	64
Benavante et al. 2021	ENA PRJEB44419	Uganda	NA	4
Malariagen4;1049-PV-VN-BONI	ENA PRJNA240356-PRJNA240533; PRJNA295233	Vietnam	Binh Phuoc	12
Malariagen4;1052-PF-TRAC-WHITE	ENA PRJNA240356-PRJNA240533; PRJNA295233	Vietnam	Binh Phuoc	1
Malariagen4;1157-PV-MULTI-PRICE	ENA PRJNA240356-PRJNA240533; PRJNA295233	Vietnam	Binh Phuoc	17
Malariagen4;1157-PV-MULTI-PRICE	ENA PRJNA240356-PRJNA240533; PRJNA295233	Vietnam	Dak O	31
Malariagen4;1128-PV-MULTI-GSK	ENA PRJNA240356-PRJNA240533; PRJNA295233	Vietnam	Ho Chi Min	42
Malariagen4;1157-PV-MULTI-PRICE	ENA PRJNA240356-PRJNA240533; PRJNA295233	Vietnam	Krong Pa	34
Pearson et al. 2016	ENA PRJEB2140	Vietnam	NA	14
Malariagen4;1049-PV-VN-BONI	ENA PRJNA240356-PRJNA240533; PRJNA295233	Vietnam	Viet Anh Ward	1

*Lo et al. Initially described 20 of their samples in this publication. In MalariaGEN's follow-up, an additional 4 were described.
